# Single-cell transcriptomics reveals peripheral immune responses in non-segmental vitiligo

**DOI:** 10.3389/fimmu.2023.1221260

**Published:** 2023-11-22

**Authors:** Pengju Yang, Mei Luan, Weizhe Li, Mengtian Niu, Qiannan He, Yixin Zhao, Jianan Chen, Binyue Mao, Kuanhou Mou, Pan Li

**Affiliations:** ^1^ Department of Dermatology, the First Affiliated Hospital of Xi’an Jiaotong University, Xi’an, China; ^2^ Center for Translational Medicine, the First Affiliated Hospital of Xi’an Jiaotong University, Xi’an, China

**Keywords:** vitiligo, single-cell RNA sequencing, immune cells, Tregs, B cells, monocytes, neutrophils

## Abstract

**Background:**

Vitiligo is a common autoimmune depigmented dermatology due to destruction of melanocytes. Much evidence suggests that vitiligo is associated with systemic immune activation. Previous studies have focused on immune cell infiltration in and around lesion areas, but few studies have investigated the cell types and function of circulating immune cells in peripheral blood. Here, single cell RNA-sequencing (scRNA-seq) was used to investigate the mechanisms of peripheral immune responses in vitiligo patients.

**Methods:**

Peripheral blood was collected from five patients with progressive non-segmental vitiligo and three healthy controls. Peripheral blood mononuclear cells (PBMCs) were obtained by Ficoll-Paque density gradient centrifugation, and scRNA-seq was performed on isolated cell populations to obtain single cell transcriptomes and characterize important genes and intracellular signaling pathways. The key findings were validated with qPCR and flow cytometry assays.

**Results:**

We identified 10 major cell types by scRNA-seq. Among these cell types, neutrophils were specifically observed in our scRNA-seq data from PBMCs. Peripheral blood effector CD8+ T cells from vitiligo patients did not show significant differences at the transcriptome level compared with healthy controls, whereas regulatory T cells showed pro-inflammatory TH1-like properties. Innate immune cells, including natural killer cells and dendritic cells, showed increased antigen processing and presentation as well as upregulated interferon responses. B cells, monocytes, and neutrophils all showed activation. B cells, especially memory B cells, had upregulated expression of genes related to humoral immunity. Monocytes showed production of proinflammatory cytokines and chemokines. Neutrophils showed strong chemokine ligand-receptor (L-R) pair (CXCR8-CXCR2) autocrine signaling pathway.

**Conclusion:**

This study revealed the genetic profile and signaling pathway characteristics of peripheral blood immune cells in vitiligo patients, providing new insights into its pathogenesis, which may facilitate identification of potential therapeutic targets.

## 1 Introduction

Vitiligo is a common depigmented dermatology caused by the loss of melanocytes from skin and mucous membranes with a global prevalence of approximately 0.5%-2% (1). It is widely accepted to be an autoimmune disease (2). Autoantibodies have been found in serum from vitiligo patients. Moreover, vitiligo patients are more likely to suffer from combined autoimmune diseases. These diseases include autoimmune thyroid disease, type 1 diabetes, rheumatoid arthritis (RA), systemic lupus erythematosus (SLE), and Addison disease (3–6).

Many previous studies have emphasized the major role of immune cells in skin and peripheral blood in the autoimmune pathogenesis of vitiligo. These immune cells include cytotoxic T lymphocytes, regulatory T cells (Tregs), dendritic cells (DCs), natural killer (NK) cells, and innate lymphoid cells. Melanocyte-specific CD8+ T cells increase in the lesions and blood of vitiligo patients and play a direct role in melanocyte destruction (7, 8). Vitiligo patients also have a decreased proportion of peripheral blood Tregs with an impaired inhibitory T cell function compared with healthy controls (HCs) (9, 10). The proportion of myeloid dendritic cells and plasmacytoid dendritic cells (pDCs) in peripheral blood is significantly increased in vitiligo patients compared with HCs (11). Myeloid dendritic cells play a critical role in proinflammatory cytokine production and autoantigen presentation (11). Infiltrated pDCs in skin lesions of vitiligo patients may produce interferon (IFN)-α associated with immune cell recruitment (12). The number of innate immune cells (NK and innate lymphoid cells) is increased in the skin and blood of vitiligo patients, and these cells are involved in the initiation of the autoimmune process of vitiligo (13). Therefore, it is necessary to study peripheral blood immune cells in patients with non-segmental vitiligo.

Single-cell RNA-sequencing (scRNA-seq) is rapidly emerging as a highly effective method to investigate gene expression heterogeneity at the single cell level. scRNA-seq is used to characterize the heterogeneity of cell populations and identify novel cell populations associated with diseases (14). Analysis of the immune cells in patients with skin diseases, such as psoriasis and atopic dermatitis, by scRNA-seq has revealed the heterogeneity of skin immune cells in these diseases, which may facilitate better understanding of the pathogenesis of inflammatory skin diseases (15). scRNA-seq has also been used to analyze the type and function of immune cells in the skin of vitiligo patients. Harris et al. collected samples of suction blister biopsies and used scRNA-seq to characterize immune cell subsets associated with the skin lesions of vitiligo and found that CCR5+ Tregs limit disease progression (16). Chen et al. found a higher proportion of CD8+ cytotoxic T lymphocytes in the skin of vitiligo patients than in normal individuals, which expressed higher levels of IFN-γ, and that fibroblasts are the predominant IFN-γ-responsive cell type in vitiligo patients by scRNA-seq analysis of skin tissue obtained by biopsy (17). The application of single-cell sequencing to skin tissue from vitiligo patients has revealed the pathogenesis of vitiligo. Systematic studies describing the functions and populations of peripheral blood immune cells in the pathological state of vitiligo as a systemic disease have been lacking. Therefore, in this study, peripheral blood immune cells of five PV patients and three HCs were collected for analysis by scRNA-seq. Cells from the PBMC layer were isolated by density gradient centrifugation and included not only peripheral blood mononuclear cells, but also a population of neutrophils defined as low density neutrophils (LDNs) (18). LDNs are a subset of neutrophils that have received the most recent attention and are generally reported to be low buoyancy neutrophil contamination in PBMCs (19). LDNs are associated with various immune-mediated inflammatory diseases such as SLE and psoriasis (20, 21). Therefore, we speculated that LDNs might be related to the pathogenesis of vitiligo.

In this study, we aimed to map circulating immune cells in vitiligo patients and evaluate the expression of key genes and intracellular signaling pathways involved in these cells. This study may provide the basis to reveal the pathogenesis of vitiligo autoimmunity and new targets to predict the disease.

## 2 Methods

### 2.1 Sample collection and preparation of single cell suspensions

Peripheral blood samples were collected from five vitiligo patients and three HCs at the Department of Dermatology of The First Affiliated Hospital of Xi’an Jiaotong University after written informed patient consent. All patients were diagnosed with progressive non-segmental vitiligo marked by enlargement of lesions or the formation of new lesions within 6 months and did not receive systemic treatment prior to enrollment. Exclusion criteria were autoimmune disease, infectious disease, cancer, and other depigmented dermatoses such as nevus anemicus, senile leukoderma, and pityriasis alba. Two milliliters of peripheral venous blood were collected from each sample into EDTA-anticoagulant tubes and diluted with 2 mL PBS. The blood was layered onto 3 mL Ficoll-Paque (GE Healthcare, Sweden) in a 10 mL centrifuge tube and centrifuged at 400 g for 30 min at 18°C. The PBMC fraction was transferred to another tube and washed twice with PBS to obtain a single cell suspension. Cell viability was required to exceed 90% for each sample. Written informed consent was obtained from the relatives of patients and healthy volunteers.

### 2.2 Single cell RNA-seq and preliminary results

PBS was added to the single cell suspension to prepare a final concentration of 1×10^5^ cells/mL. The final single cell suspension was loaded onto a microfluidic device for the Singleron GEXSCOPE Single Cell RNA Library Kit (Singleron Biotechnologies) in accordance with the manufacturer’s protocol, which involved cell lysis, mRNA capture, labeling cells (barcode) and mRNA (UMI), reverse transcription of mRNA into cDNA and amplification, and cDNA fragmentation. Individual libraries were diluted to 4 nM and pooled for sequencing. Pools were sequenced on an Illumina HiSeq X with 150 bp paired-end reads.

### 2.3 Primary analysis of raw read data

CeleScope^®^ was employed to process raw data, using double-ended FASTQ files as input and generating files and QC metrics for downstream data analysis. Briefly, sequencing data containing a cell barcode (CB) and a unique molecular identifier (UMI) were read. After filtering the read without poly-T tails, valid CBs and UMIs were extracted and used to calculate the expression level of each gene in each cell. FastQC and fastp were used to process raw reads to remove low sequencing quality reads, and poly-A tails and adaptor sequences were removed using cutadapt. After quality control, STAR Genome Mapper was applied to map the extracted reads to the reference genome GRCh38 (ensembl version 92 annotation). UMI counts and gene counts for each cell were obtained using featureCounts v1.6.2 software. Sequencing reads from the same gene, CB, and UMI were pooled together as PCR repeats and used to generate expression matrix files for subsequent analysis.

### 2.4 Quality control, dimension reduction, and clustering

Seurat package (v3.1.2) was used for quality control (QC), dimension reduction, and clustering (22). Cells with gene counts below 200, the top 2% gene counts, and the top 2% of UMI counts were filtered out. Then, cells with >20% mitochondrial content were eliminated. After QC, the remaining cells were used for further analysis. All gene expression was normalized and scaled using NormalizeData() and ScaleData(). We selected the top 2000 variable genes using the FindVariableFeautres() function and then applied PCA. Principal component analysis was set to 1:20 for unsupervised cell clustering. To reduce potential batch effects, samples were integrated and downstream analysis was performed using Harmonity v0.1. Cells were clustered by FindClusters() using the top 20 principle components with a resolution parameter of 2.0. The uniform manifold approximation and projection (UMAP) algorithm was applied to visualize cells in two dimensions.

### 2.5 Differentially expressed gene analysis and cell type annotation

Genes expressed in >10% of cells in a cluster with an average log (fold change) of >0.25 were selected as DEGs using the FindAllMarkers function in Seurat v3.1.2 based on the Wilcox likelihood ratio test with default parameters. Using the SynEcoSys database containing >8800 marker genes, the cell type identity of each cluster of peripheral blood circulating immune cells was determined by the expression of typical marker DEGs. Heat maps, dot plots, and violin plots were generated using Seurat v3.1.2 DoHeatmap, DotPlot, and Vlnplot, respectively, to visualize the expression of markers used to identify each cell type. The final results were manually checked to ensure correctness and visualized by UMAP.

### 2.6 Pathway enrichment analysis

ClusterProfler R Package v4.0.2 was applied to analyze Gene Ontology (GO) to study the potential functions of each cell subset (23). The gene ontology of gene sets included molecular function (MF), biological process (BP), and cellular component (CC) categories as references. Significantly enriched pathways were those with p_adj-values < 0.05. Gene set enrichment analysis (GSEA) of the HALLMARK-INFLAMMATORY-RESPONSE pathway in neutrophils was performed using the irGSEA R package, and calculation of the enrichment scoring method was set to AUCell.

### 2.7 Trajectory analysis and SCENIC analysis

To map the differentiation of cell subtypes, pseudotime trajectory analysis was performed with Monocle2 (24). We used DDRTree and the FindVariableFeatures to perform dimension reduction. Finally, the trajectory was visualized by the plot_cell_trajectory. Furthermore, to assess transcription factor (TF) regulation strength, we applied the single-cell regulatory network inference and clustering (pySCENIC, version 0.11.1) workflow (25).

### 2.8 Cell–cell communication analysis

R language-based CellChat software package was used to calculate cell–cell interactions expressed by known ligand–receptor pairs in various cell types (26). We used gene expression data from cells and the specified cell type as input to CellChat. Subsequent analysis was performed using the ligand–receptor database in CellChat, and computeCommunProb and computeCommunProPathway were used to calculate the possible interactions and pathways. Hierarchical, circular, and chordal plots were used to visualize signaling pathways.

### 2.9 Flow cytometry assays

PBMCs were isolated from peripheral blood of five vitiligo patients and five HCs. To determine the level of NK cells and HLA-DR expression, the following antibodies were used for surface staining: anti-CD3-PE, anti-CD56-APC, anti-CD16-FITC, and anti-HLA-DR-PE/Cyanine7(all from BioLegend, San Diego, USA). PBMCs were cultured in RPMI 1640 medium containing 100 U/mL penicillin, 100 U/mL streptomycin, and 10% fetal bovine serum. The cell density was approximately 1×10^6^/mL. Cell Stimulation Cocktail (eBioscience, San Diego, USA) and GolgiPlug Protein Transport Inhibitor (BD Biosciences Pharmingen, San Jose, USA) were added to the medium, and cells were then incubated for 5 h at 37°C in 5% CO2. For Tregs and Th1-like Tregs detection, cells were surface stained with anti-CD4-FITC, anti-CD25-APC, anti-CD127-PE (all from BioLegend, San Diego, USA), and then intracellular staining for anti-IFN-gamma-PE/Cy7(BD Biosciences Pharmingen, San Jose, USA) was performed using Cytofix/Cytoperm fixation/permeabilization kit (BD Biosciences Pharmingen, San Jose, USA) according to the manufacturer’s instructions. Flow cytometry analysis was performed using NovoCyte 3000 (ACEA Biosciences, San Diego, USA). Data analysis was performed using NovoExpress software (ACEA Biosciences, San Diego, USA).

### 2.10 Quantitative real-time PCR

Total RNA was extracted from PBMCs using BioZol reagent (BIODEE, Beijing, China) according to the manufacturer’s instructions. Reverse transcription was performed on 1μg of RNA from each sample using the Evo M-MLV RT Mix Kit with gDNA Clean for qPCR (Accurate Biotechnology, Hunan, China). qPCR was performed on the Bio-Rad CFX-96 Real-Time PCR System (Bio-Rad, Hercules, CA, USA) using the SYBR Green Premix Pro Taq HS qPCR Kit (Accurate Biotechnology, Hunan, China). Each PCR was replicated three times for verification, and the 2-ΔΔCt method was used to analyze the relative changes in gene expression from the qPCR experiments. Glyceraldehyde-3-phosphate dehydrogenase (GAPDH) was used as a reference gene in all qPCR experiments. The following primer pairs were used for the qPCR: FoxP3, forward: 5’-TTTCTGTCAGTCCACTTCACCAA-3’ and reverse: 5’-TTCAAGGAAGAAGAGGAGGCATG-3’; CTLA-4, forward: 5’-ATGGACACGGGACTCTACATCTG-3’ and reverse: 5’-GGGCACGGTTCTGGATCAATTA-3’; CXCL8, forward: 5’-TAGGACAAGAGCCAGGAAGAAAC-3’ and reverse: 5’-GGGTGGAAAGGTTTGGAGTATGT-3’; CXCL9, forward: 5’-TGATTGGAGTGCAAGGAACCC-3’ and reverse: 5’-AATTTTCTCGCAGGAAGGGCT-3’; CCL5, forward: 5’-TCGCTGTCATCCTCATTGCTACT-3’ and reverse: 5’-CACTTGCCACTGGTGTAGAAATAC-3’; CXCR2, forward: 5’-GCAACCCAGGTCAGAAGTTTCAT-3’ and reverse: 5’-TCAAAGCTGTCACTCTCCATGTT-3’.

### 2.11 Bulk transcriptomic data obtained from the GEO database

Expression profiling was performed using publicly available raw data (accession number GSE80009) in R (version 4.3.0). GEOquery and limma R packages from GEO2R were used to identify the differential expression of all samples. GO analysis was performed by clusterProfiler package in R software. The cut-off criteria for DEGs were log2 fold change (FC) > 1 and adjusted P value < 0.05.

### 2.12 Statistics

All statistical analyses were performed with GraphPad Prism 8.0 software. Unpaired Student’s t-test and Mann-Whitney U-test were used to determine significance. A P-value of less than 0.05 was considered statistically significant.

## 3 Results

### 3.1 Single cell atlas of circulating immune cells from PV patients and HCs

**Figure 1:**
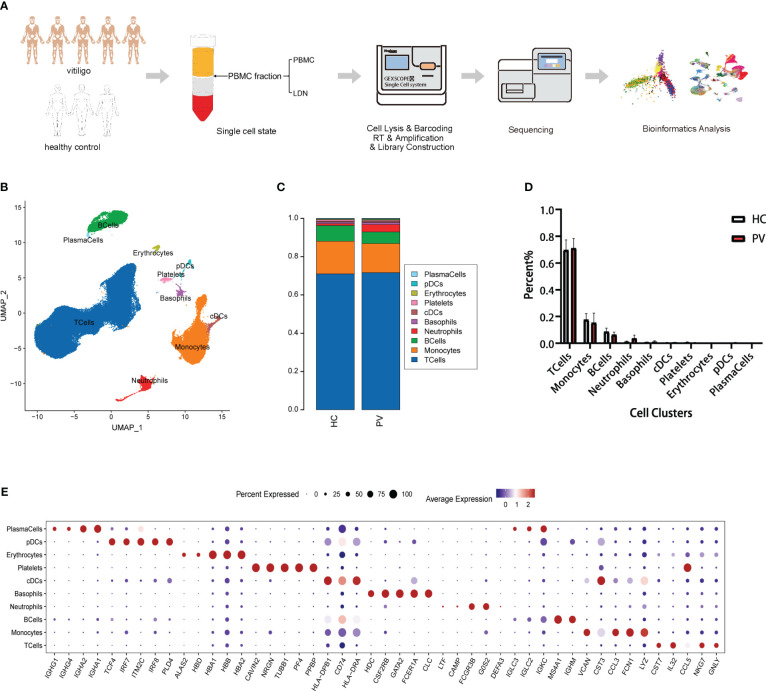
Single-cell landscape of circulating immune cells from PV patients and HCs. **(A)** Schematic flowchart of scRNA-seq experimental design of this study. **(B)** UMAP representation of 71,966 and 55,838 single cells from PV patients (n = 5) and HCs (n = 3), respectively, showing the formation of 10 clusters. **(C)** The fraction of cells for ten cell types in HCs and PV patients. **(D)** Bar graphs of each cell cluster population between HCs and PV patients. **(E)** Bubble plot of top5 gene expression in each cell cluster; the size of the bubble represents the percentage of expressed cells; the color represents the average expression of each gene in clusters: red means the high expression.

We collected the PBMC layer after density gradient centrifugation from five patients diagnosed with PV and three HCs, which was subjected to scRNA-seq to characterize the peripheral immune microenvironment of PV patients (**Figure 1A**). Clinical information on the five patients and three HCs in our study is shown in **Supplementary Table 1**. At a sequencing depth of ~30,000 reads/cell, a median of 530–1,326 genes per cell were detected in patients and 866–1,356 genes per cell in HCs (**Supplementary Table 2**). After identifying and removing low quality cells with a low unique molecular identifier (UMI) or high mitochondrial RNA, we obtained 71,966 and 55,838 individual transcription profiles from PV patients and HCs, respectively. After confirming that the data integration removed residual batch effects, the data from different samples showed excellent reproducibility (**Supplementary Figure 1**). Thus, in accordance with the expression of typical marker genes, we identified 10 major cell types (**Figure 1B**). Among the cell populations were NK and T cells (CD3D+TRAC+), monocytes (FCN1+CD14+), B cells (CD79A+MS4A1+), plasma cells (CD79A+IGHG1+), classical dendritic cells (cDCs; CD1C+CD1E+), plasmacytoid DCs (pDCs; LILRB4+CLEC4C+), and platelets (PPBP+GP9+), basophils (CLC+CPA3+), erythrocytes (HBA1+HBB+), and neutrophils (CAMP+ CSF3R+) (**Figure 1E**, **Supplementary Figure 2**). The proportions of the 12 cell types were compared between PV patients and HCs (**Figure 1C**). No significant differences were found in the composition of the cells between the two groups (**Figure 1D**). Platelets, erythrocytes, and basophils were discarded from the subsequent analysis. To explore differences in peripheral blood immune cells between PV patients and HCs, we performed DEG and pathway enrichment analyses.

### 3.2 Characteristics of T and NK cells in PV patients

**Figure 2:**
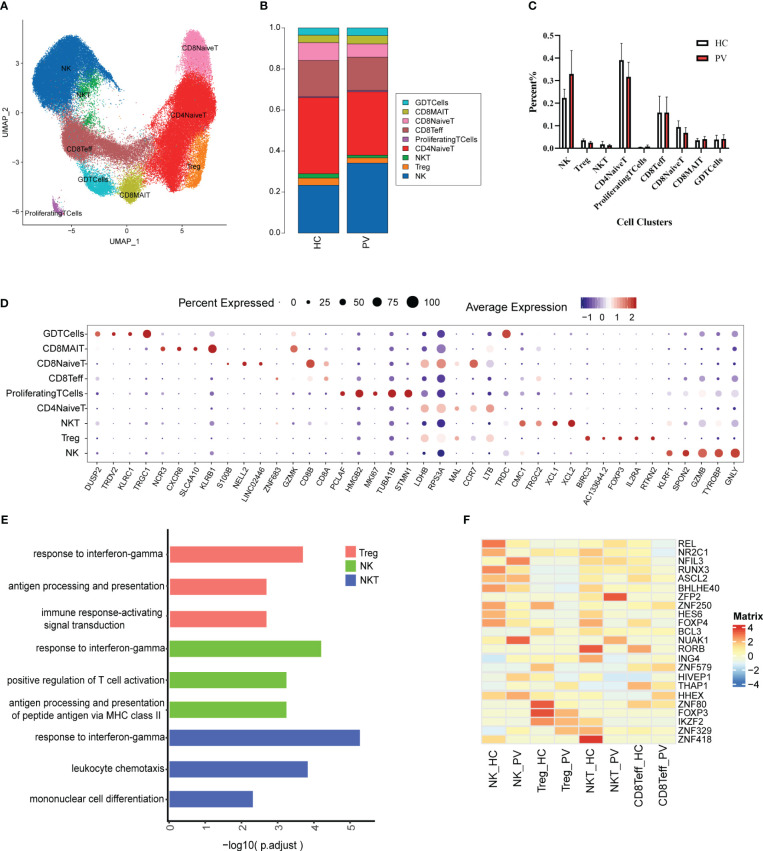
The heterogeneity and transcriptional features of NK&T cells in PV patients. **(A)** UMAP representation of 91,224 NK&T cells, showing the formation of nine clusters. **(B)** The fraction of cells for nine types in HCs and PV patients. **(C)** Bar graphs of each cell cluster population between HCs and PV patients. **(D)** Bubble plot of top5 gene expression in each cell cluster; the size of bubble represents the percentage of expressed cells; the color represents the average expression of each gene in clusters: red means the high expression. **(E)** GO analysis showing the biological process enriched in Treg NK and NKT of PV patients. **(F)** Heatmap showing the average expression of key regulatory TFs (estimated using SCENIC) between NK cells and T cell subsets in PV patients and HCs.

T and NK cells are the most abundant immune cells among peripheral blood mononuclear cells. We classified the 91,224 NK and T cells into nine subtypes (**Figure 2A**) using classical NK and T cell markers (**Supplementary Figure 3**): NK cells, naive CD4+ T cells (CD4NaiveT), naive CD8+ T cells (CD8NaiveT), effector CD8+ T cells (CD8Teff), CD8+ mucosa-associated constant T cells (CD8MAIT), γδ T cells (GDTcells), NKT cells, regulatory T cells (Tregs), and proliferating T cells (ProliferatingTCells). The relative percentages of the various cell subtypes were calculated for each patient. The proportion of NK cells in the peripheral blood of vitiligo patients was increased compared with that in HCs, while the proportion of Tregs was lower than in HCs, but this difference was not statistically significant (**Figures 2B, C**). Furthermore, there were no significant differences in the proportions of other subpopulations between HCs and PV patients (**Figures 2B, C**). The expression levels of representative DEGs were compared in NK and T cells between HCs and PV patients before cell subtype annotation (**Figure 2D**). Thirty-five genes were significantly upregulated, including GZMB, CTSW, MIF, ALOX5AP, and other genes related to cell killing and the inflammatory response, and 15 genes were downregulated, including KLF2, TXNIP, and LEF1 (**Supplementary Figure 5A**). We further analyzed the differences in T cell subsets. DEGs were characterized in CD4NaiveT, CD8NaiveT, CD8Teff, CD8MAIT, GDTcells, NKT cells, and Tregs. scRNA-seq revealed differential expression of 36 genes in CD4NaiveT, 38 genes in CD8NaiveT, 41 genes in CD8Teff, 39 genes in CD8MAIT, 58 genes in GDTcells, 113 genes in NKT cells, and 48 genes in Tregs (**Supplementary Table 3**).

**Figure 3:**
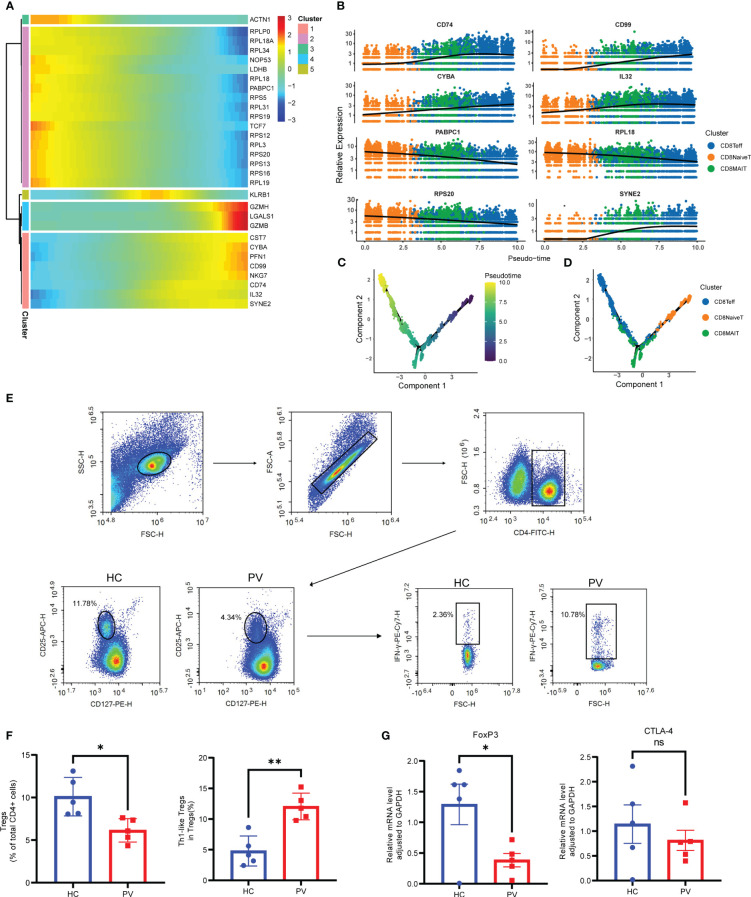
Analysis of CD8+ T cells differentiation trajectories; flow cytometry and qPCR validation of Tregs and Th1-like Tregs. **(A)** Heatmap showing the dynamic changes of gene expression along pseudotime. **(B)** The trajectory of six typical genes. Unsupervised transcriptional trajectory from Monocle, colored by pseudotime **(C)** and cell clusters **(D)**. **(E)** Representative gating strategy of flow cytometry analysis for proportion of Tregs (CD4+CD25+CD127low/-) in CD4+ T cells and Th1-like Tregs (IFN-γ+ Treg) in Tregs. **(F)** Flow cytometry analysis of Tregs and Th1-like Tregs. **(G)** Analysis of FoxP3 and CTLA-4 mRNA expression by qPCR. Data are expressed as mean ± SEM and significance was set at *p ≤ 0.05, **p ≤ 0.01.

To further analyze functional differences in T cell subsets, we performed pathway enrichment analysis of DEGs in NK and T cells. No significant differences were found in GO analysis of naive CD4+ T cells, naive CD8+ T cells, CD8+ mucosa-associated constant T cells, and γδ T cells (**Supplementary Table 4**). Tregs possess functional plasticity, and Tregs upregulate T-BET expression upon IFN-γ stimulation in a STAT1-dependent manner, and additional or subsequent exposure to IL-12 induces Tregs to adopt a TH1-like phenotype, express T-BET and release IFN-γ (27, 28). We found that Tregs were involved in the up-regulatory response to interferon-γ (**Figure 2E**). Therefore, we detected the composition of Tregs, Th1-like Tregs and the expression of key genes by flow cytometry and qPCR. Flow cytometry analysis revealed PV patients had a reduced abundance of circulating Tregs, but an increased proportion of Th1-like Tregs compared with HCs (**Figures 3E, F**). Since the expression of FoxP3 and CTLA-4 is critical for the immunosuppressive function of Tregs (29), we investigated the expression of FoxP3 and CTLA-4 mRNA in PBMC by qPCR. Our results revealed that FoxP3 gene expression was significantly decreased in the peripheral blood of PV patients compared with HCs, while CTLA-4 gene expression was decreased, although not significantly different (**Figure 3G**). NK cells were involved in antigen processing and presentation, upregulated responses to interferon, and T cell activation. Because HLA-DR-expressing NK cells combine phenotypic characteristics of NK cells and dendritic cells with antigen-presenting ability (30), we verified the percentage of HLA-DR-expressing NK cells in the peripheral blood of PV patients by flow cytometry and found that it was increased compared with HCs, but there was no statistically significant difference (**Supplementary Figure 6**). NKT cells were involved in upregulated responses to interferon, regulation of chemotaxis, and antigen processing and presentation (**Figure 2E**). These results suggested that NK and T cells of PV patients, especially NK and NKT cells, exhibited consistent inflammatory responses such as upregulation of responses to interferons.

Subsequently, given the stronger association of NK, Treg, NKT, and Teff with PV, we employed single-cell regulatory network inference and clustering (SCENIC) to provide a more comprehensive analysis of the TFs that exhibited abnormal activation in these four cell types among PV patients and HCs (**Figure 2F**). The expression of REL, an important regulator of the anti-inflammatory factors IL-12 and IL-10 signaling, and RUNX3, a regulator that inhibits myeloid cell differentiation, as well as several unknown regulators (NR2C1, BHLHE40, ZNF250, HES6, and FOXP4) were downregulated in NK cells from PV patients compared with HCs; we also found upregulation of the expression of NFIL3, an important TF for NK cell differentiation, and other regulators (NUAK1, HIVEP1, HHEX). The Treg stability and function related regulators FOXP3, IKZF2 and other regulators (BCL3, ZNF579, ZNF80) were significantly downregulated in Tregs from PV patients. The expression of RORB, ING4, IKZF2, ZNF418 were downregulated in NKT from PV patients compared with HCs, in contrast to the expression of ZFP2, NUAK1. In CD8Teff, the expression of ASCL2, a suppressor of Th1, Th2 and Th17 cell differentiation, was downregulated, whereas the expression of HHEX was upregulated.

Next, we performed pseudotime trajectory analysis on CD8+ T cells. The heatmap depicted the difference in the gene expression of CD8+ T cells from CD8NaiveT to CD8MAIT and CD8Teff (**Figure 3A**). With the direction of pseudotime, CD8+ T cells gradually shifted from cluster 2 genes with the high expression of PABPC1, RPS20 and RPL18 to cluster 1 and cluster 4 with the high expression of GZMH and NKG7, respectively (**Figure 3B**). The results of cell trajectory analysis showed no differences between samples (**Supplementary Figures 8A, B**). CD8NaiveT was considered to be a precursor for other cells, and the composition of the cell subpopulation developed from CD8NaiveT to CD8MAIT and CD8Teff (**Figures 3C, D**).

### 3.3 Immunological characteristics of B cell subsets in PV patients

**Figure 4:**
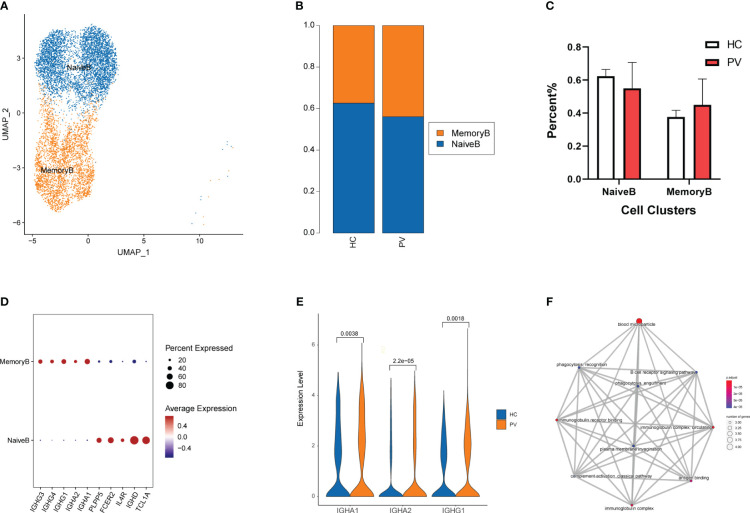
The heterogeneity and transcriptional features of B cells in PV patients. **(A)** UMAP representation of B cells, showing the formation of two clusters. **(B)** The fraction of cells for ten two types in HCs and PV patients. **(C)** Bar graphs of each cell cluster population between HCs and PV patients. **(D)** Bubble plot of top 5 gene expression in each cell cluster; the size of the bubble represents the percentage of expressed cells; the color represents the average expression of each gene in clusters: red means the high expression. **(E)** The violin diagram shows the expression levels of IGHA1, IGHA2 and IGHG1 in PV patients and HCs. **(F)** The mapplot of GO pathways of the DEGs in memory B cells of PV patients.

To analyze changes in B cell subsets in the peripheral blood of vitiligo patients, we labeled B cell subsets by their known typical genes and classified them into two cell subtypes: naive and memory B cells (**Figure 4A**, **Supplementary Figures 4A, B**). Next, we evaluated differences in the distribution of peripheral blood B cell subpopulations between HCs and PV patients (**Figure 4B**). The proportion of memory B cells in peripheral blood was increased in PV patients compared with HCs, but there was no significant difference between the two groups. Moreover, no significant differences in cell proportions were found in naive B cells (**Figure 4C**). By comparing the transcriptional profiles of naive and memory B cells, we identified representative marker genes of each cell cluster of B cells. Naive B cells expressed TCL1A, IGHD, and FCER2 at higher levels, whereas memory B cells expressed IGHG1, IGHG3, and IGHG4 at higher levels (**Figure 4D**). Next, we compared changes in DEGs for each cell subpopulation of B cells between PV patients and HCs. **Supplementary Table 5** shows the details of the B cells and two subpopulations of significant DEGs in PV patients and HCs. **Supplementary Table 6** shows the details of the GO enrichment analysis results. DEG analysis revealed that PTMAP2, MT-TW, MT-RNR1, MT-ND4L, MTND2P28, MTCO1P12, MTATP6P1, and HBA2 were overexpressed in naive and memory B cells from PV patients (**Supplementary Figure 5B**, **Supplementary Table 5**). Additionally, three upregulated differentially expressed genes were involved in humoral immunity, IGHA1, IGHA2 and IGHG1, in memory B cells of PV patients (**Figure 4E**). We also evaluated the functional characteristics of B cell subsets (**Supplementary Table 6**). In memory B cells, GO enrichment analyses (**Figure 4F**) showed that DEGs participated in B cell activation, such as the immunoglobulin complex and B cell receptor signaling pathway, suggesting that adaptive immunity was involved in PV.

### 3.4 Features of monocytes and DCs in PV patients

**Figure 5:**
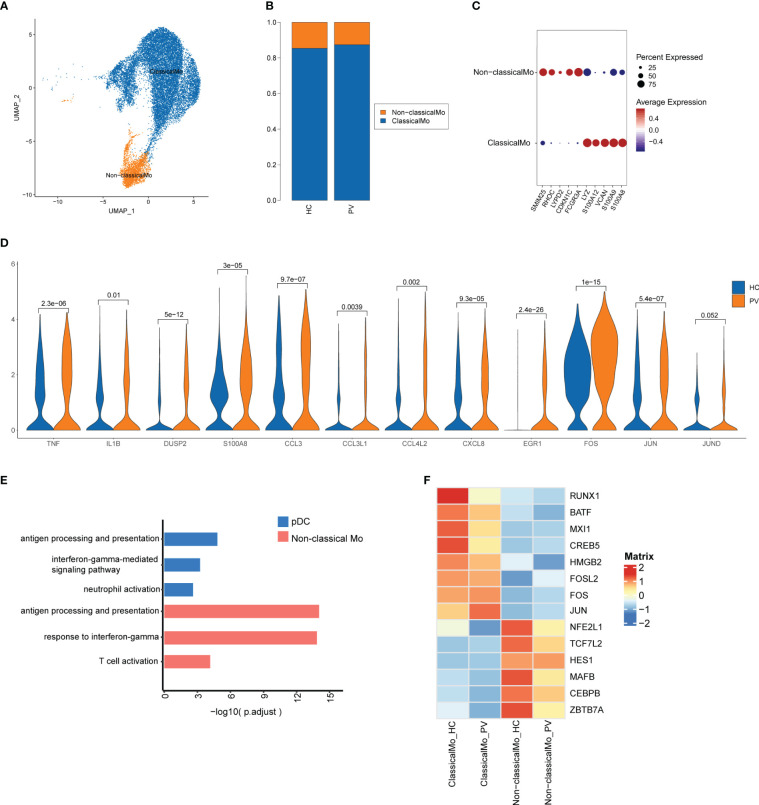
The heterogeneity and transcriptional features of monocytes in PV patients. **(A)** UMAP representation of monocytes, showing the formation of two clusters. **(B)** The fraction of cells for two types in HCs and PV patients. **(C)** Bubble plot of top5 gene expression in each cell cluster; the size of the bubble represents the percentage of expressed cells; the color represents the average expression of each gene in clusters: red means the high expression. **(D)** The violin diagram shows the expression levels of TNF, IL1B, DUSP2, S100A8, CCL3, CCL3L1, CCL4L2, CXCL, EGR1, FOS, JUN, and JUND in PV patients and HCs. **(E)** GO analysis showing the biological process enriched in non-classical monocytes and pDC of PV patients. **(F)** Heatmap showing the average expression of key regulatory TFs (estimated using SCENIC) between monocyte subsets in PV patients and HCs.

Next, we further investigated transcriptome changes in two more prevalent subsets of innate immune cells, namely monocytes and DCs. We analyzed 17,877 monocytes and found 9,632 cells originated from PV patients and 8,245 originated from HCs (**Supplementary Table 2**). Monocytes were further labeled in the classical annotation by CD14 and FCGR3A to obtain two cell subpopulations: classical (CD14 high) and non-classical [CD14 low, FCGR3A(CD16) high] monocytes (**Figure 5A**). The relative proportions of each cell subtype were calculated in each patient (**Figure 5B**). Marker genes are displayed for each subset in **Supplementary Figures 4C, D**. We next characterized representative marker genes in each monocyte population. Classical monocytes expressed high levels of S100A8, S100A9, VCAN, S100A12, and LYZ, while non-classical monocytes expressed high levels of SMIM25, RHOC, LYPD2, CDKN1C, and FCGR3A (**Figure 5C**). To explore the variation in monocytes between PV patients and HCs, we compared DEGs across cell subpopulations. Considering that classic monocytes represent a higher proportion of monocytes, we found that classical monocytes and unclassified cells exhibited similar characteristics. Expression of human leukocyte antigen (HLA)-related genes, such as HLA-DRB5, HLA-DPA1, and HLA-DPB1, was significantly higher in peripheral blood monocytes of PV patients compared with HCs, suggesting increased antigen processing and presentation in the peripheral blood monocytes of PV patients (**Supplementary Figure 5C**, **Supplementary Table 7**). The top GO terms of classical monocytes from PV patients were elevated, including cellular response to xenobiotic stimulus and transport vesicle membrane (**Supplementary Table 8**). Non-classical monocytes in the peripheral blood of PC patients had clear upregulated expression of inflammatory genes (TNF, IL1B, DUSP2, and S100A8), chemokines (CCL3, CCL3L1, CCL4L2, and CXCL8), and TFs (EGR1, FOS, JUN, and JUND) compared with HCs (**Figure 5D**). Using GO functional enrichment analysis, we found that significant DEGs in non-classical monocytes from PV patients were involved in the response to interferon-gamma, antigen processing and presentation, and T cell activation (**Figure 5E**). For example, in non-classical monocytes from PV patients, T cell activation genes were strongly upregulated, including HLA-DPA1, HLA-DPB1, CD74, EGR1, HLA-DRA, HLA-DRB1, HLA-DMB, ZFP36L1, and IL1B. We verified that FOS and HLA-DRB5 expression was upregulated in PBMC from vitiligo patients compared with HCs by analyzing Bluk-RNA seq data from GEO database (**Supplementary Figure 7A**).

To further investigate the transcriptional differences between classical and non-classical monocytes, we analyzed their TF regulators using SCENIC (**Figure 5F**). Classical monocytes preferentially upregulated RUNX1, MXI1, HMGB2, and AP-1 TFs (CREB5, BATF, FOSL2, FOS, and JUN) (more active in PV: FOS and JUN; more active in HC: RUNX1, BATF, MXI1 and CREB5), whereas non-classical monocytes upregulated TFs including NFE2L1, TCF7L2, HES1, MAFB, CEBPB, and ZBTB7A, which were more active in HCs.

In addition to monocytes, DCs are another major group of innate immune cells. We analyzed 801 cDCs (486 cells from PV patients and 315 cells from HCs) and 491 pDCs (312 cells from PV patients and 179 cells from HCs) (**Supplementary Table 2**). The relative proportions of each cell subtype were calculated in each patient (**Figure 1D**). The proportions of each subgroup were not significantly different between PV patients and HCs. **Figure 1E** shows the marker genes of each subset. We next examined the transcriptome characteristics of these two subgroups. We compared the gene expression of cDCs in peripheral blood of PV patients and HCs. We found 31 significantly upregulated genes and six significantly downregulated genes among the differentially expressed genes between cDCs of the two groups of peripheral blood (**Supplementary Figure 5D**, **Supplementary Table 9**). We found that significantly DEGs were involved in antigen processing and presentation PV patients by GO analysis (**Supplementary Table 10**). Moreover, we found 180 significantly upregulated genes and 174 significantly downregulated genes among the differentially expressed genes between the two groups of peripheral blood pDCs (**Supplementary Figure 5E**). The DEGs were related to antigen processing and presentation, neutrophil degranulation, and the interferon-gamma-mediated pathway (**Figure 5E**, **Supplementary Table 10**).

Both monocytes and DCs demonstrated an upregulated response to interferon-gamma and antigen processing and presentation. These results indicated that innate immune cells of PV patients, especially non-classical monocytes and pDCs, exhibited an active inflammatory state.

### 3.5 Immunological features of neutrophils in PV patients

**Figure 6:**
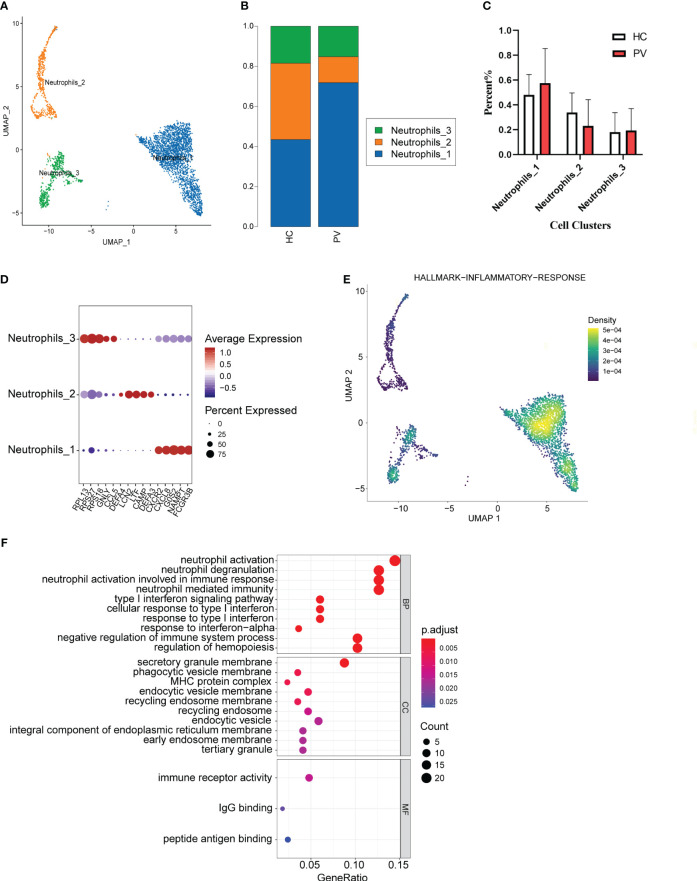
The heterogeneity and transcriptional features of neutrophils in PV patients. **(A)** UMAP representation of neutrophils, showing the formation of three clusters. **(B)** The fraction of cells for three types in HCs and PV patients. **(C)** Bar graphs of each cell cluster population between HCs and PV patients. **(D)** Bubble plot of top5 gene expression in each cell cluster; the size of the bubble represents the percentage of expressed cells; the color represents the average expression of each gene in clusters: red means the high expression. **(E)** UMAP plot of distribution of inflammatory response pathway in neutrophils. **(F)** The dotplot of GO pathways of the DEGs in Neutrophils_3 of PV patients.

We analyzed the features of neutrophil subsets in PBMCs, also known as LDNs, to reveal the heterogeneity of these cells in PV patients. Mechanical compartmentalization divided neutrophils into three populations named Neutrophils_1 with differentially expressed FCGR3B, CXCL8, and SAT1, Neutrophils_2 with upregulated DEFA3, CAMP, and LTF, and Neutrophils_3 with differentially expressed CCR5, GNLY, and TRBC2 (**Figures 6A, D**). We compared the number of Neutrophils_2 and Neutrophils_3 of PV patients versus HCs, which also did not show significant differences (**Figure 6B**). The percentage of Neutrophils_1 in peripheral blood was higher in PV patients than in HCs, but there was no significant difference (**Figure 6C**).

To further explore the distinct changes in the neutrophil transcriptome of PV patients and HCs, we compared the gene expression profiles of PV patients and HCs, and found that DEGs in PV patients included 301 upregulated and 306 downregulated genes compared with HCs (**Supplementary Figure 5F**, **Supplementary Table 11**). DEGs upregulated in neutrophils, especially Neutrophils_3, in the peripheral blood of patients were mainly involved in neutrophil activation, T cell activation, and immune response activation signaling (**Figure 6F**, **Supplementary Table 12**). To investigate the inflammatory level in peripheral blood neutrophils, neutrophil_1 showed a higher enrichment score in GSEA (**Figure 6E**).

### 3.6 Cell interactions in circulating immune cells from peripheral blood of PV patients

**Figure 7:**
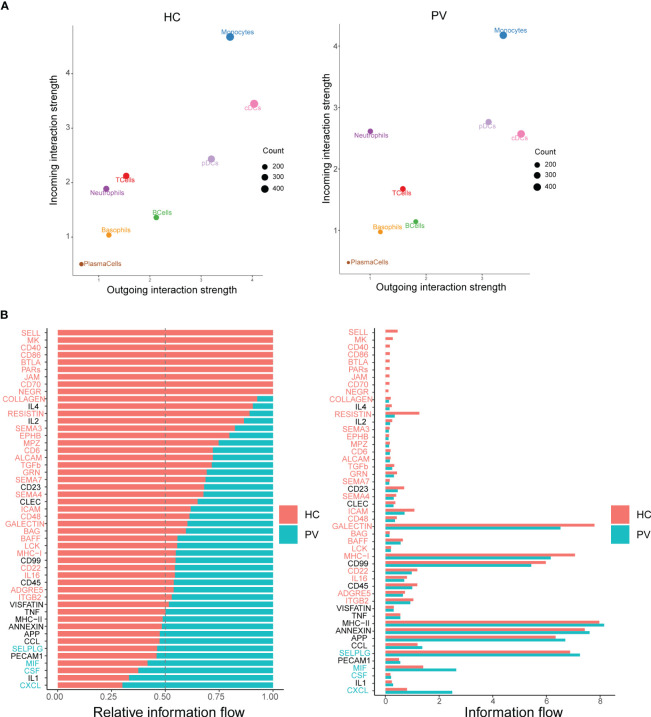
Differential communication patterns in HCs and PV patients. **(A)** Scatter plot of incoming and outgoing interaction strength of each cell population in HCs and PV patients. **(B)** Significant signaling pathways were ranked based on differences in the overall information flow within the inferred networks between HCs and PV patients. The signaling pathways depicted in red are enriched in HCs, and those depicted in green were enriched in PV patients.

Cellular interactions between immune cells are strongly associated with the progression of autoimmune diseases. CellChat infers cell–cell interactions by assessing the gene expression of receptor–ligand pairs in various cell types. We therefore used CellChat to predict the number and strength of interactions between various immune cell populations in the peripheral blood of PV patients and HCs. cDCs and neutrophils were the main affected sources and targets in PV patients compared with HCs. However, we concentrated on intercellular communication in PV patients, and the overall analysis of intercellular communication between PV patients and HCs was similar (**Figure 7A**). We identified 31 important signaling pathways by CellChat in HCs, such as TGFb, ITGB2, ICAM, and RESISTIN signaling pathways, while four pathways predicted by CellChat, including MIF, SELPLG, CSF, and CXCL pathways, were specific to circulating immune cells in the peripheral blood of PV patients (**Figure 7B**).

**Figure 8:**
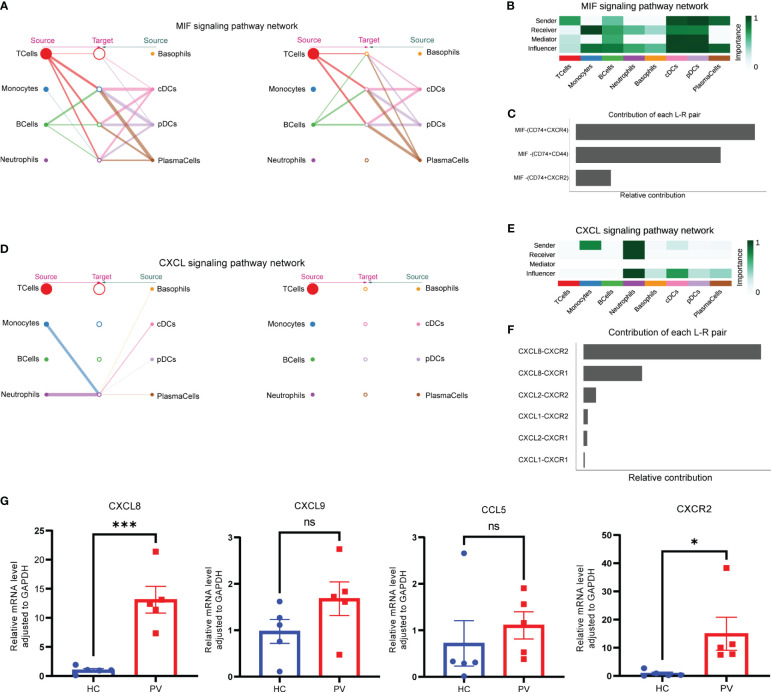
Complex intercellular communication network in the circulating immune cells in PV patients. **(A)** Hierarchical plot showing the inferred intercellular communication network for MIF signaling pathway. Circle sizes are proportional to the number of cells in each cell group and edge width represents the communication probability. **(B)** Heatmap showing the relative importance of each cell group for the four network centrality measures-based MIF signaling network. **(C)** Relative contribution of each ligand–receptor pair to the overall communication network of MIF signaling pathway. **(D)** The inferred CXCL signaling pathway network. **(E)** The computed network centrality measures of CXCL signaling. **(F)** Relative contribution of each CXCL ligand–receptor pair. **(G)** Analysis of CXCL8, CXCL9, CCL5 and CXCR2 mRNA expression by qPCR. Data are expressed as mean ± SEM and significance was set at *p ≤ 0.05, ***p ≤ 0.001.

To better understand intercellular communication, we used CellChat to study changes in MIF and CXCL signaling pathways. Macrophage migration inhibitory factor (MIF) is an important multifunctional cytokine that promotes immune cell activation and the production of other proinflammatory cytokines. Significantly elevated serum MIF levels and MIF mRNA expression have been found in vitiligo patients, suggesting that MIF is involved in the pathogenesis of vitiligo. **Figure 8A** shows the intercellular communication network between various cell clusters of the MIF pathway. The results indicated a close relationship between most immune cells via the MIF pathway. CellChat visualization of network centrality scores indicated that most outward signaling from T cells and DCs was received by monocytes, suggesting that most MIF interactions between cells were paracrine (**Figure 8B**). Notably, DCs also play an important role as a major mediator of the MIF pathway, suggesting their role as gatekeepers. Almost all cell populations are influencers of the MIF pathway. In terms of ligand–receptor pairs, the MIF pathway involves three ligand–receptor pairs. In order of relative contribution from highest to lowest, these ligand–receptor pairs were CD74–CXCR4, CD74–CD44, and CD74–CXCR2, as shown in **Figure 8C**, and they played an important role in vitiligo.

In contrast to the MIF signaling pathway, a ligand–receptor hierarchy plot of the CXCL signaling pathway showed that only one type of target cell (neutrophils), and neutrophils and monocytes were the main source (**Figure 8D**). Moreover, network centrality analysis confirmed that the neutrophils were prominent influencer cells in the CXCL signaling pathway (**Figure 8E**). Neutrophils are the major CXCL signaling source in both autocrine and paracrine pathways. In the ligand–receptor pair analysis, the CXCL signaling pathway included six ligand–receptor pairs. In order of relative contribution from highest to lowest, these ligand–receptor pairs were CXCL8–CXCR2, CXCL8–CXCR1, CXCL2–CXCR2, CXCL1–CXCR2, CXCL2–CXCR1, and CXCL1–CXCR1 (**Figure 8F**). Next, we analyzed the differences in mRNA expression of CXCL8 and CXCR2, which were found to be differentially expressed in this study, as well as CCL5 and CXCL9, which were the most studied in previous studies, in PBMCs from PV patients and HCs. The results showed that CXCL8 and CXCR2 mRNA expression was significantly increased in PBMCs from PV patients; in addition, CXCL9 and CCL5 mRNA expression was similarly increased, but not statistically different (**Figure 8G**).

Collectively, the interaction analyses highlighted the role of peripheral blood circulating immune cells in vitiligo patients in promoting immune cell hyperactivation through MIF and CXCL signaling pathways.

## 4 Discussion

By performing scRNA-seq with its high resolution, we generated a comprehensive profile of various cell types and gene expression in the peripheral blood of PV patients. This study provides a clear insight into the autoimmune mechanisms of vitiligo. Additionally, a better understanding of the functions and mechanisms of immune cell subsets in progressive vitiligo may be crucial to discover potential therapeutic targets.

**Figure 9:**
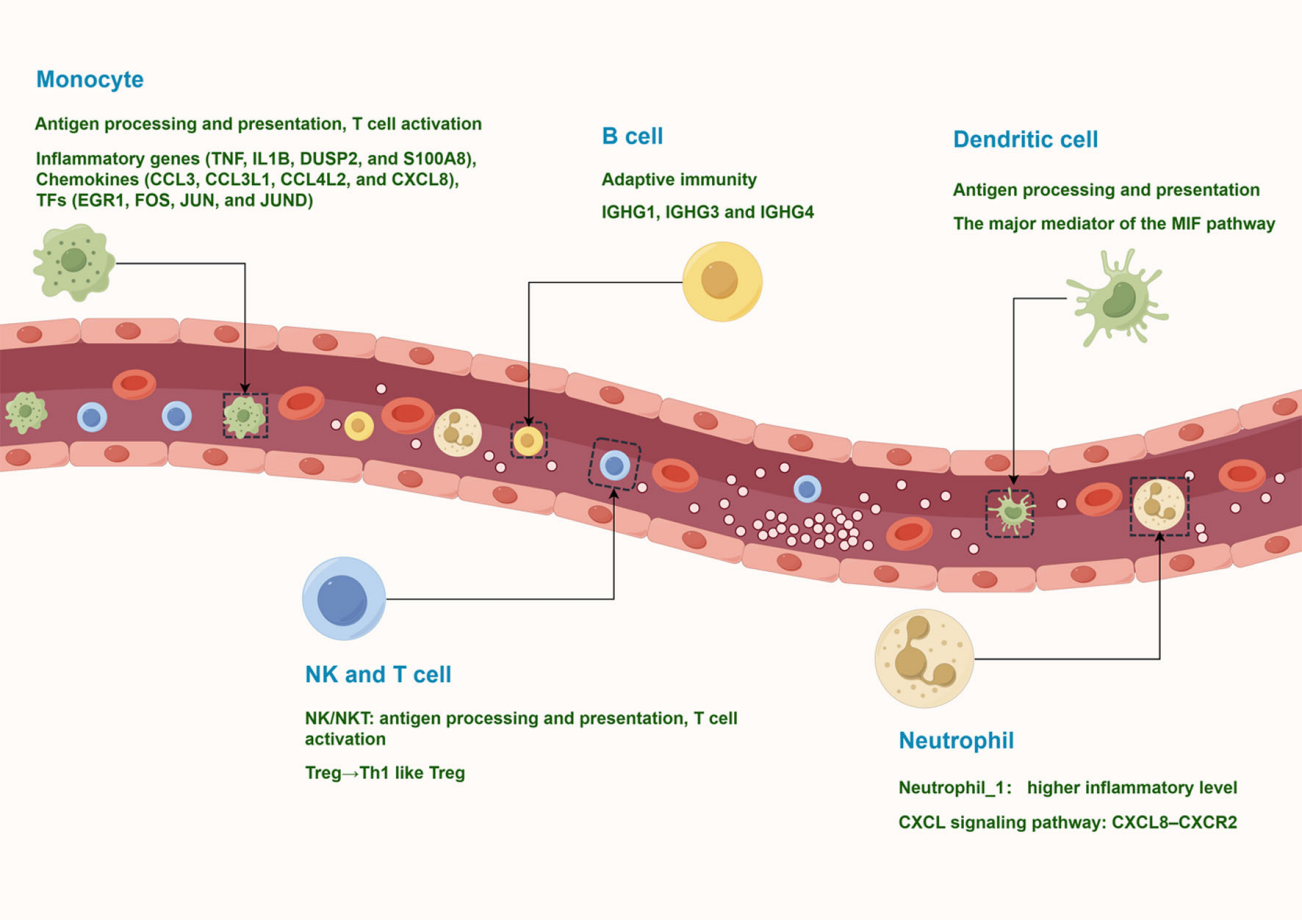
A model of the complex and heterogeneous peripheral immune microenvironment of progressive non-segmental vitiligo.

The circulating immune environment of PV patients is more complex than previously thought. To date, studies have relied on flow cytometry to analyze the major types of circulating immune cells in PV patients. However, flow cytometry relies on known markers for labeling and lacks systematic knowledge of the composition of peripheral blood immune cells. In this study, we obtained the transcriptional profiles of 127,804 immune cells by scRNA-seq and identified 10 major immune cell subtypes, such as NK and T cells, monocytes, B cells, plasma cells, classical dendritic cells, plasmacytoid DCs, and neutrophils. These cells have various states of gene expression and function (**Figure 9**). Our study revealed that peripheral blood effector CD8+ T cells from vitiligo patients did not show significant differences at the transcriptome level compared with HCs, whereas regulatory T cells showed pro-inflammatory TH1-like properties. Neutrophils showed significant CXCR8-CXCR2 autocrine signaling pathway.

A higher inflammatory response was observed in PV patients compared with HCs. We obtained similar results by analyzing bulk transcriptomics data from the GEO database, suggesting that DEG is involved in immune response-regulating signaling pathway and activation of immune response (**Supplementary Figures 7B, C**). This may explain why lesions progress more rapidly in some PV patients. However, bulk transcriptomic data cannot reveal cell type-specific expression profiles, obscuring the heterogeneity of cell subpopulations, whereas scRNA-seq overcomes this deficiency and analyzes the composition and transcriptional status of circulating immune cells. T cells were grouped into seven clusters, including effector CD8+ T cells, NKT cells, and Tregs. Previous studies have indicated that cytotoxic T lymphocytes play a central role in the pathogenesis of vitiligo, infiltrating vitiligo lesions to specifically kill melanocytes (31). Additionally, these cells produce IFN-γ, which stimulates chemokine secretion in keratinocytes and promotes T cell recruitment to lesions (32). However, this study revealed that peripheral blood effector CD8+ T cells did not have significant differences at the transcriptome level, suggesting that effector CD8+ T cells are activated to kill melanocytes after recruitment to skin tissue. Tregs are another predominant cell population involved in the pathogenesis of vitiligo. Tregs promote peripheral immune tolerance by inhibiting the activation, expansion, and cytokine secretion of CD4+ and CD8+ T cells through the secretion of inhibitory cytokines and cell–cell contacts (28). The number of Tregs in vitiligo patients is reduced or unaltered, and defective and dysfunctional Tregs in vitiligo patients expand and activate CD8+ T cells (33–35). However, in our study, peripheral blood Tregs from vitiligo patients showed proinflammatory rather than immunosuppressive properties, including upregulation of the response to interferon. Similar results were obtained by Li et al. who recently reported that Tregs from vitiligo patients show a Th1-like phenotype and impaired inhibition of CD8+ T cell proliferation and activation (10). In addition to T cell-mediated cellular immunity, another important component of the adaptive immune response involved in vitiligo is B cell-mediated humoral immunity. This study supports the idea that vitiligo is characterized by an increase in antibody-secreting B cells, especially memory B cells. Serum levels of melanocyte-specific antibodies are significantly elevated in PV patients and correlate to disease activity, indicating systemic immune activation in vitiligo patients, which is consistent with our study (36). In vitiligo patients, innate immune cells, such as NK cells and DCs, are abnormally activated. NK cells have been implicated in the early development of vitiligo as a potential initiator of T cell autoreactivity (13). NK cells are innate effector cells that produce IFN-γ and are the first defense line against viral infections and malignant cells. Although previous studies have found increased circulating NK cells in the peripheral blood of vitiligo patients, little was known about their role in vitiligo development until recently (37, 38).

By analyzing the scRNA-seq data, we found that NK and NKT cells exhibited different characteristics in PV patients compared with HCs, and were involved in increased antigen processing and presentation, upregulated responses to interferon, and T cell activation in PV patients. One study has shown that patients with vitiligo have a significantly lower proportion of invariant NKT (iNKT) cells, a subpopulation of peripheral NKT cells (39). Although iNKT cells make up only a small fraction of lymphocytes, their ability to rapidly secrete a large number of cytokines, including IFN-γ, IL-4, IL-10, and IL-13, makes them important regulators of the Th1/Th2 cytokine balance in the immune response (39). Studies on the role of iNKT cells in the pathogenesis of inflammatory skin diseases such as atopic dermatitis, psoriasis, and allergic contact dermatitis suggest that the impact of iNKT cells is dichotomic, that is, protective or pathogenic (40). We hypothesized that the decrease in the percentage of NKT cells in peripheral blood and the altered phenotype suggest that NKT cells play an immunomodulatory role in the pathogenesis of vitiligo. DCs are well-defined antigen-presenting cells in the immune response and regulate activation of T cells (41, 42). DCs interact with various immune cell types, such as CD8+, CD4+, Th17, and NK cells, to produce a variety of proinflammatory cytokines such as IFN-γ and TNF-α. DCs are involved in the pathogenesis of autoimmune diseases such as psoriasis, RA, SLE, Sjögren’s syndrome and diabetes mellitus (43). Recent studies have shown that numbers of two subsets of circulating DC, namely mDCs and pDCs, are higher in PV patients than in HCs (16). pDCs to infiltrate the lesions of vitiligo patients and produce type I interferons to drive activation and recruitment of autoimmune T cells (12). The authors found that pDCs in PV patients are involved in antigen processing and presentation, neutrophil degranulation, and interferon-gamma-mediated signaling pathways.

Among innate immune cell types, monocytes are the least studied in vitiligo. A previous study reported a significant increase in the proportion of CD80+ monocytes in peripheral blood of vitiligo patients compared with HCs, suggesting that monocytes are involved in vitiligo development, but their function has not been investigated (37). Here, we identified distinct expression profiles and functional differences in monocytes from PV patients. DEG analysis showed that inflammatory cytokine, chemokine, and transcription factor genes were highly expressed in monocytes from PV patients. Interestingly, non-classical monocytes from PV patients upregulated the expression of inflammatory protein-related genes such as TNF, CCL3, and NLRP3. Therefore, monocytes from PV patients exhibit activated and proinflammatory characteristics that promote the inflammatory state by releasing proinflammatory cytokines and chemokines.

Neutrophils are the most abundant subpopulation of leukocytes in peripheral circulation and play a crucial role in defense against invading pathogens and responses to sterile inflammation, although they have a short lifespan (44). They produce ROS, secrete proteases, and release neutrophil extracellular traps as the first responders to infection and inflammation (45). Furthermore, neutrophils may behave as antigen-presenting cells and produce proinflammatory cytokines and chemokines. Recently, neutrophils were found to communicate with and regulate almost all immune cells in inflammatory responses (46). Neutrophils are associated with several skin diseases, including psoriatic inflammation and pruritus in atopic dermatitis, but few studies have focused on neutrophils in vitiligo (47, 48). Although neutrophil chemoattractant CXCL8 is highly expressed in vitiligo lesions, neutrophils are absent (49). However, the ratio of neutrophils to lymphocytes is considerably increased in PV patients compared with HCs, and ROS production in neutrophils is significantly higher than in HCs (50, 51). Because LDNs were isolated together with PBMCs during density gradient centrifugation, most neutrophils identified in our PBMC scRNA-seq data were LDNs. Abnormal increases in LDNs have been reported in many autoimmune diseases, and significant heterogeneity in LDN functional responses appears to be associated with disease (52). There is a strong suggestion that these cells play a role in the pathogenesis of autoimmune diseases by enhancing the pr-inflammatory response, altering phagocytosis, and enhancing the ability to synthesize type I interferons (19). Additionally, these cells are prone to form neutrophil extracellular traps, which may promote autoantigen externalization and organ damage (19). This study showed that the percentage of Neutrophils_1 exhibiting proinflammatory properties was higher in vitiligo patients than in controls, although not statistically significant. Pathway enrichment analysis suggested that neutrophils in vitiligo patients manifested an activated state. CellChat analysis of the CXCL signaling pathway suggested that neutrophils were a main source of CXCL and also receivers, suggesting that neutrophils exert significant autocrine signaling. The above results indicate that neutrophils may contribute to the development of vitiligo, especially maintaining the inflammatory state of peripheral blood.

Human leukocyte antigen (HLA) class II molecules are essential to generate anti-infective immune responses and are highly polymorphic, recognizing a wide variety of antigens. Several autoimmune diseases are associated with strong HLA-associated genetic background and the presentation of endogenous antigens by HLA (53). Both HLA class I and II regions are closely associated with the pathogenesis of vitiligo. Perilesional melanocytes express HLA-DR and present antigens that promote autoreactive T cells to recognize and attack melanocytes (53). Interestingly, we found increased expression of HLA class II in most peripheral blood immune cells from PV patients. In particular, HLA class II genes, such as HLA-DRB5, HLA-DPA1, and HLA-DPB1, were significantly upregulated in monocytes from PV patients. Additionally, the MIF receptor CD74 is an important MHC class II chaperone that regulates antigen presentation for the autoimmune response (54). CellChat network centrality analysis of the MIF pathway revealed that MIF ligands from T cells and DCs were predominantly taken up by monocytes. MIF upregulates the production of proinflammatory cytokines. It also induces chemotactic migration and maintains the survival of activated monocytes/macrophages (55). Therefore, we hypothesized that T cells secreted MIF to promote monocyte activation and proinflammatory cytokine secretion to maintain the inflammatory state of the peripheral immune microenvironment in vitiligo patients.

This study had several limitations. First, eight participants were included in this study, which is a relatively small sample size. Second, coupled with the fact that scRNA-seq is not sensitive to statistical differences between cell populations, no differences in the composition of individual cell subpopulations were found between PV patients and HCs. Although our study identified changes in genes and pathways, such as chemokine networks, in immune cells found in peripheral blood, further experiments are necessary to validate the underlying mechanisms. There were also limitations in the sample source for this study. Because of the difficulty in obtaining skin tissue from lesions of PV patients, the study relied on peripheral blood, which limited the study of cellular interactions between skin tissue and peripheral blood.

In summary, we revealed the peripheral blood immune landscape of PV patients by scRNA-seq, identifying changes in gene and pathway profiles compared with HCs. We found that PV patients are characterized by regulatory T cells, NK cells, and DCs with proinflammatory properties and activated B cells, monocytes, and neutrophils. Our results may facilitate exploration of vitiligo pathogenesis and the development of potential therapeutic targets.

## Data availability statement

The original contributions presented in the study are publicly available. This data can be found here: https://www.ncbi.nlm.nih.gov/geo/ under the accession number GSE231794.

## Ethics statement

The studies involving humans were approved by The Ethics Committee of the First Affiliated Hospital of Xi’an Jiaotong University. The studies were conducted in accordance with the local legislation and institutional requirements. The participants provided their written informed consent to participate in this study.

## Author contributions

PY and ML designed the study, performed the literature search, data analysis, data interpretation, and statistical analysis, and drafted of the manuscript. WL isolated PBMCs by density gradient centrifugation for scRNA-seq. MN and QH participated in the discussion. YZ, JC, and BM helped with patient recruitment. KM and PL designed the study, funded the research, provided patient samples, and drafted the manuscript. All authors contributed to the article and approved the submitted version.
