# Supplementary material for: Single-cell transcriptomics reveals peripheral immune responses in non-segmental vitiligo
Source: Front Immunol. 2023 Nov 22;14:1221260. doi: 10.3389/fimmu.2023.1221260 (PMC10702986; doi:10.3389/fimmu.2023.1221260)
Supplement: Supplementary file 1 [file DataSheet_1.docx]

Supplementary Material

**Single-Cell Transcriptomics Reveals Peripheral Immune Responses in Non-Segmental Vitiligo**

**Pengju Yang^1†^, Mei Luan^1†^, Weizhe Li^1^, Mengtian Niu^1^, Qiannan He^1^, Yixin Zhao^1^, Jianan Chen^1^, Binyue Mao^1^, Kuanhou Mou^1*^, Pan Li^2*^**

*** Correspondence:**Kuanhou Mou
mkhn001@163.com
Pan Li
imlipan@163.com

# Supplementary Figures

**
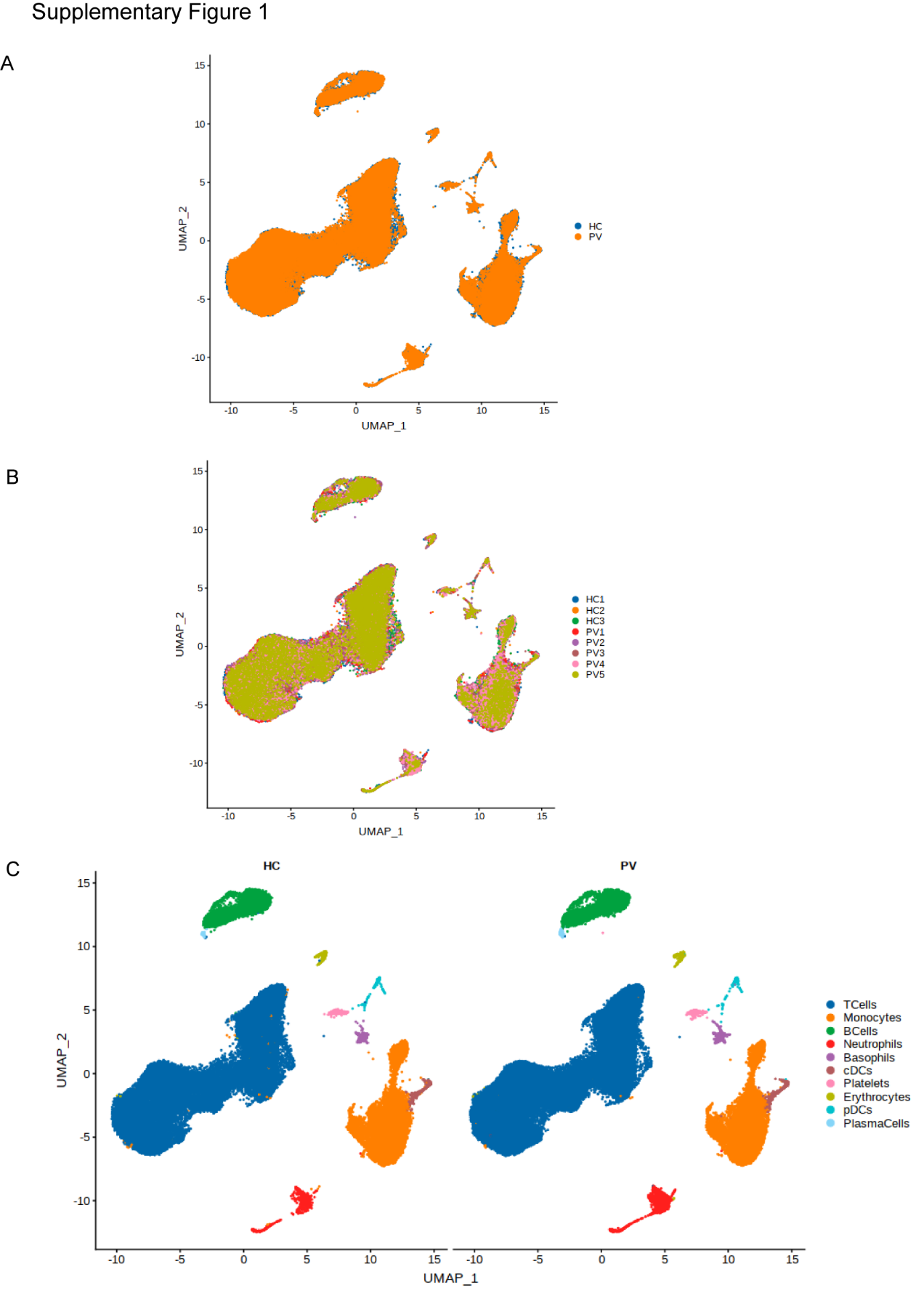
**

**Supplementary Figure 1.** UMAP dimensional-reduction projection analysis of samples from HCs (n=3) and the PV group (n=5), (A) stained by cell type source, (B) stained by group source, and (C) UMAP representation of each group

**
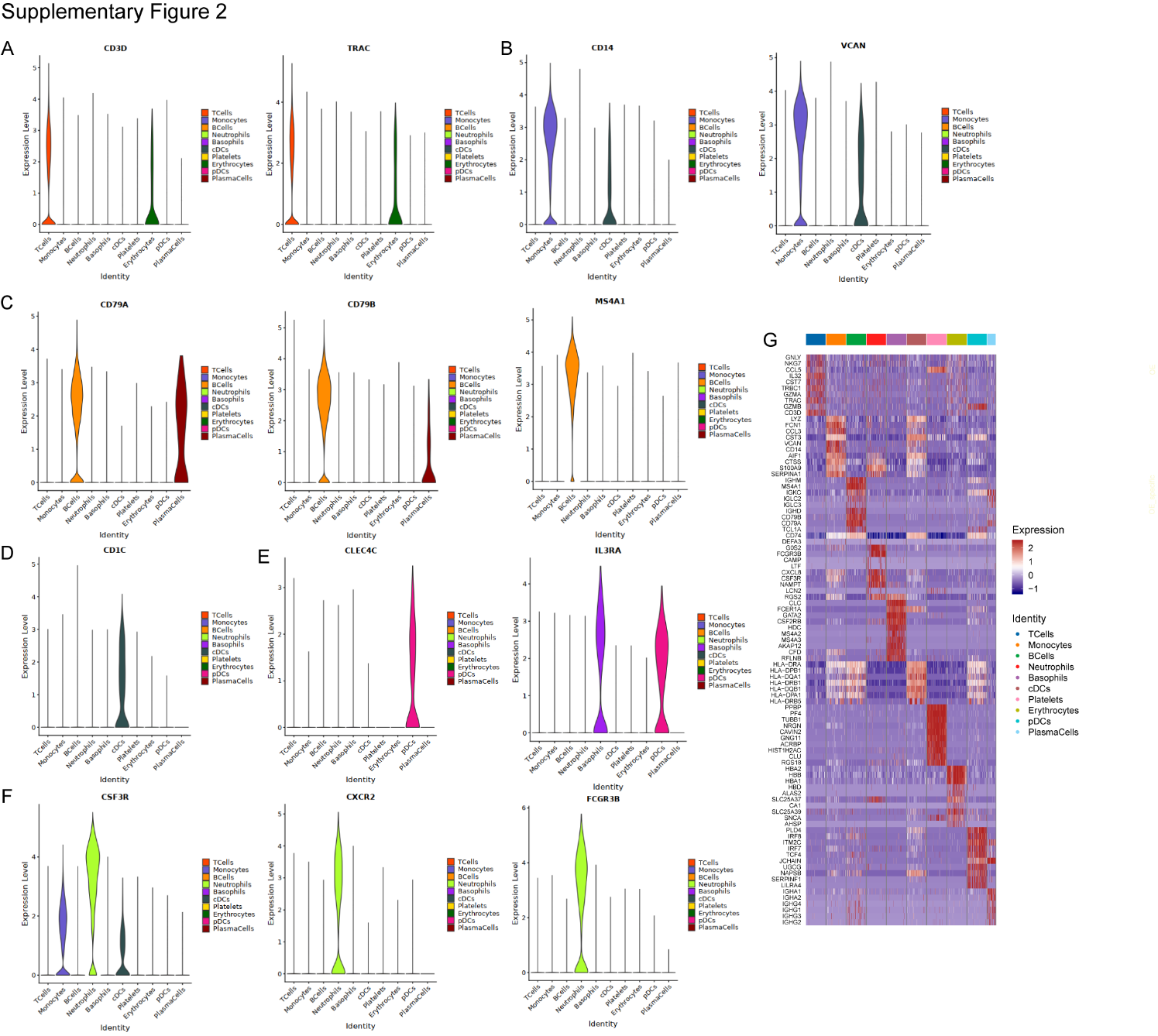
**

**Supplementary Figure 2.** Violin plot and heatmap (G) depicting the average expression and percentage of expressed cells of selected marker genes in (A)T&NK cells, (B)B cells, (C) monocytes, (D) cDCs, (E)pDCs and (F) neutrophils

**
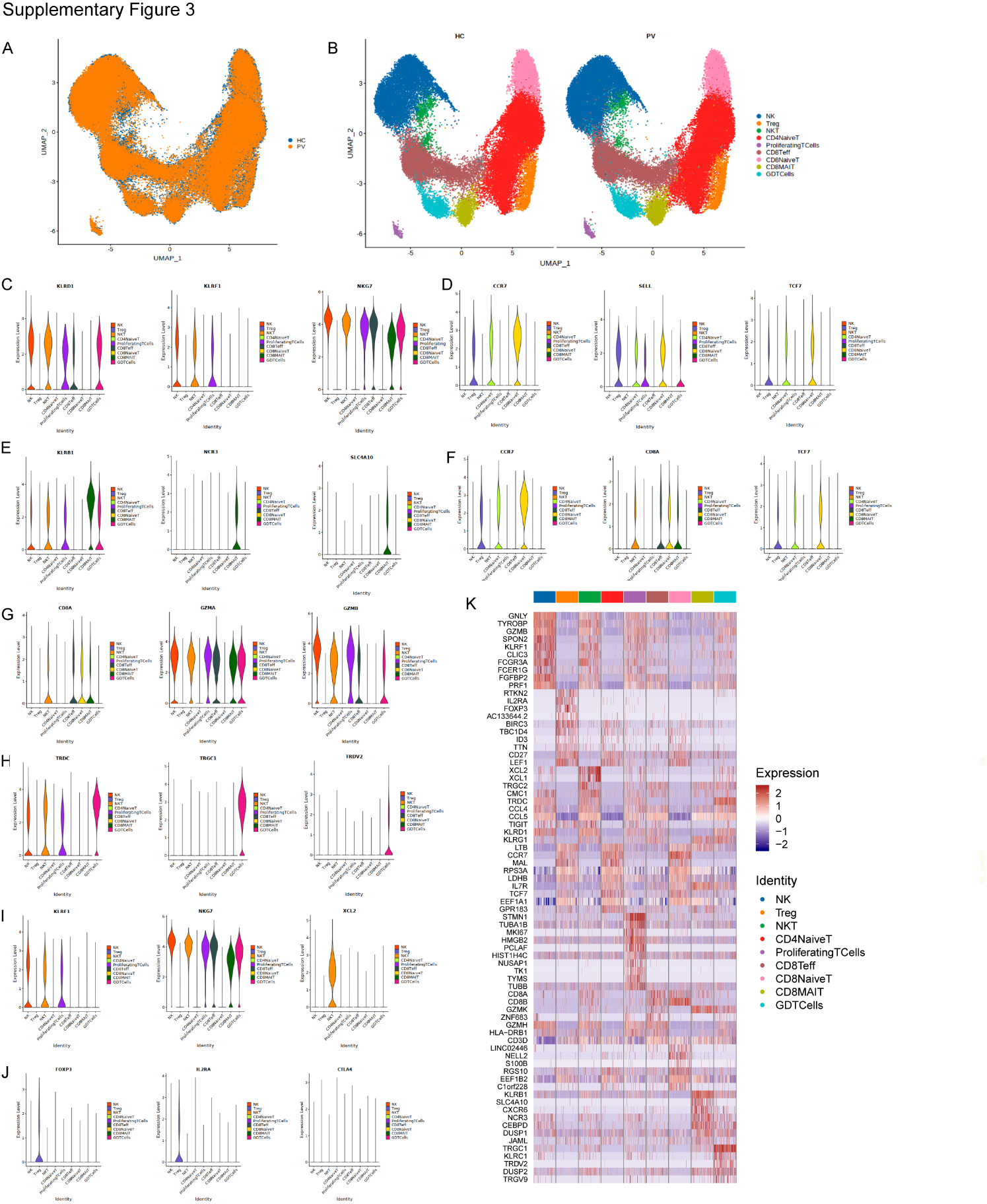
**

**Supplementary Figure 3.** UMAP dimensional-reduction projection analysis of samples from HCs (n=3) and the PV group (n=5), (A) stained by group source, (B) stained by sample source. Violin plot (C-J) and heatmap (K) depicting the average expression and percentage of expressed cells of selected marker genes in each T cell subset and NK cell

**
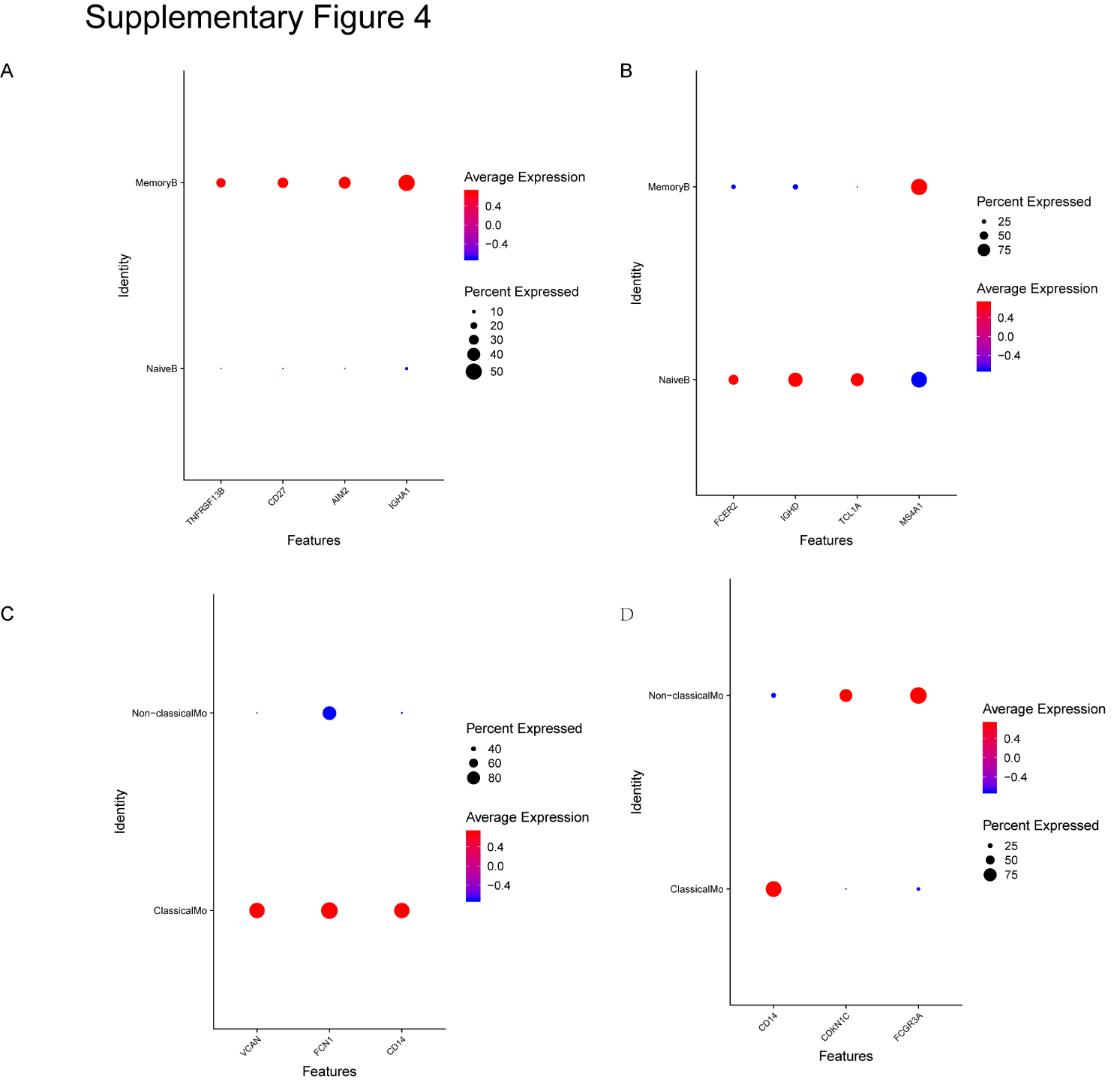
**

**Supplementary Figure 4.** Dot plot depicting the average expression and percentage of expressed cells of selected marker genes in each (A, B) B cell subsets and (C, D) monocyte subsets

**
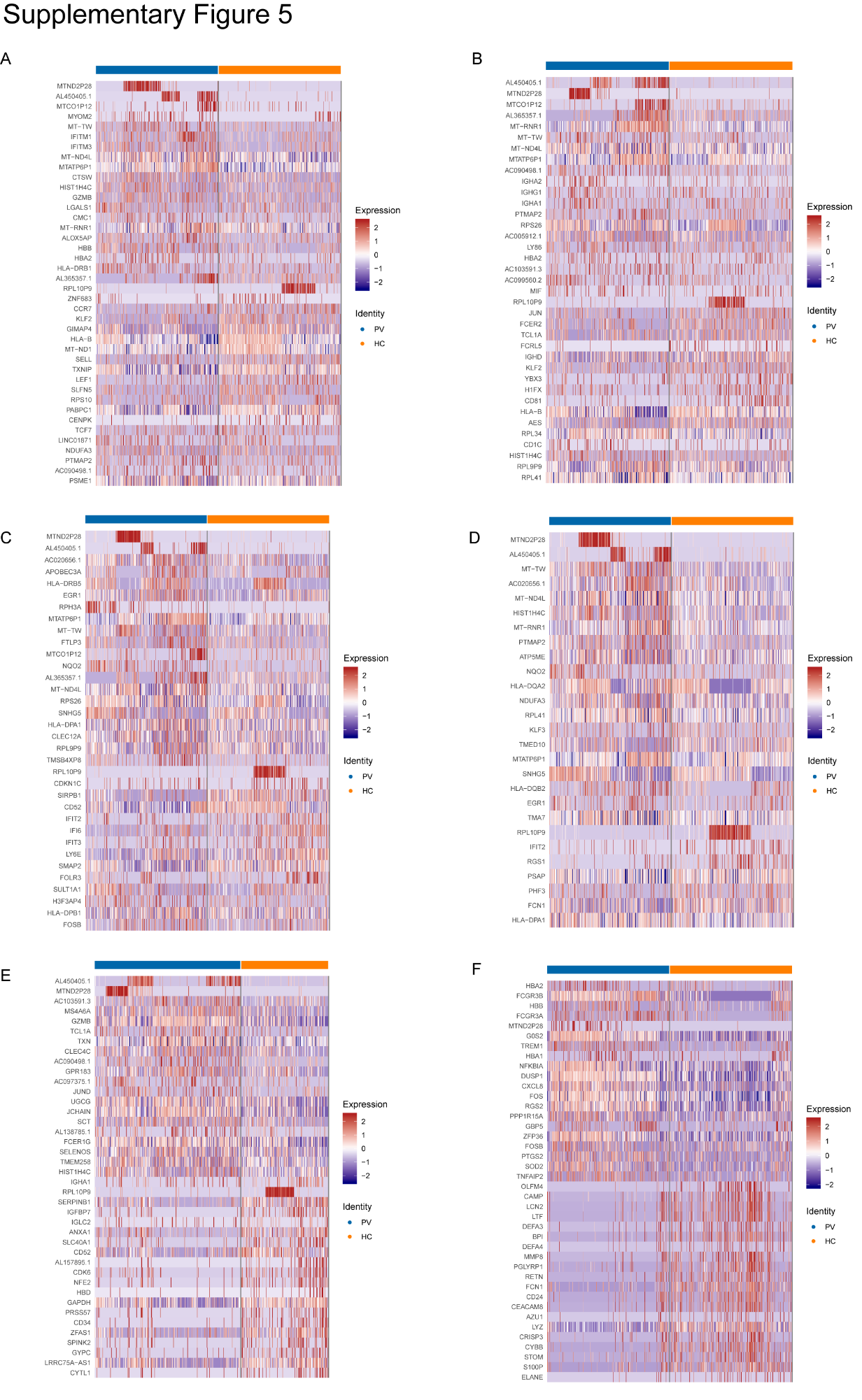
**

**Supplementary Figure 5.** Heat map of differential genes expression in (A)T&NK cells, (B)B cells, (C) monocytes, (D) cDCs, (E)pDCs and (F) neutrophils


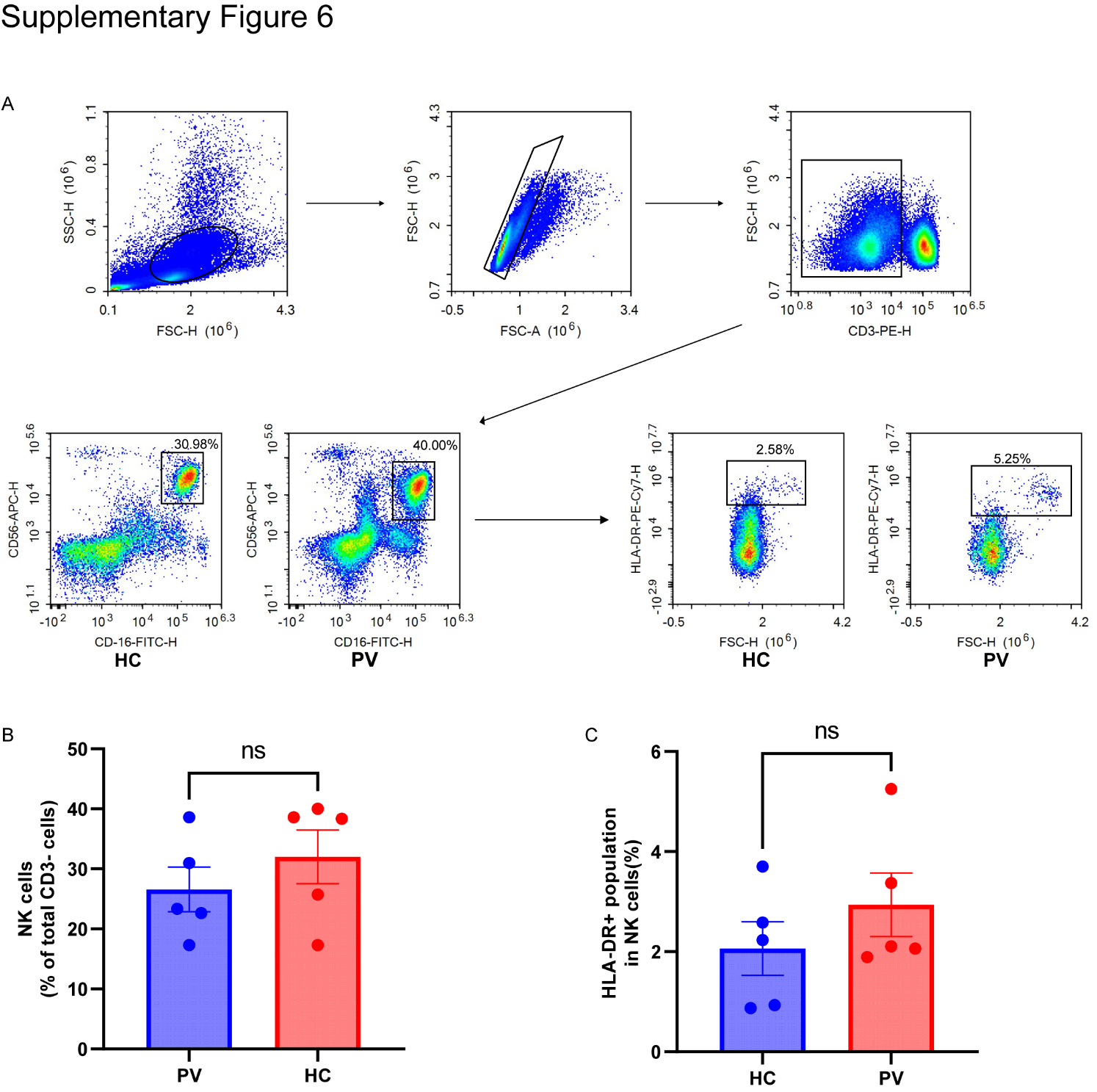


**Supplementary Figure 6.** Flow Cytometry of Tregs and Th1-like Tregs. (A)Representative gating strategy of flow cytometry analysis for proportion of NK cells in CD3- cells and HLA-DR+ NK cells in NKcells. Flow cytometry analysis of NK cells (B) and HLA-DR+ NK cells(C). Data are expressed as mean ± SEM and significance was set at *p≤0.05, **p≤0.01, ***p≤0.001.


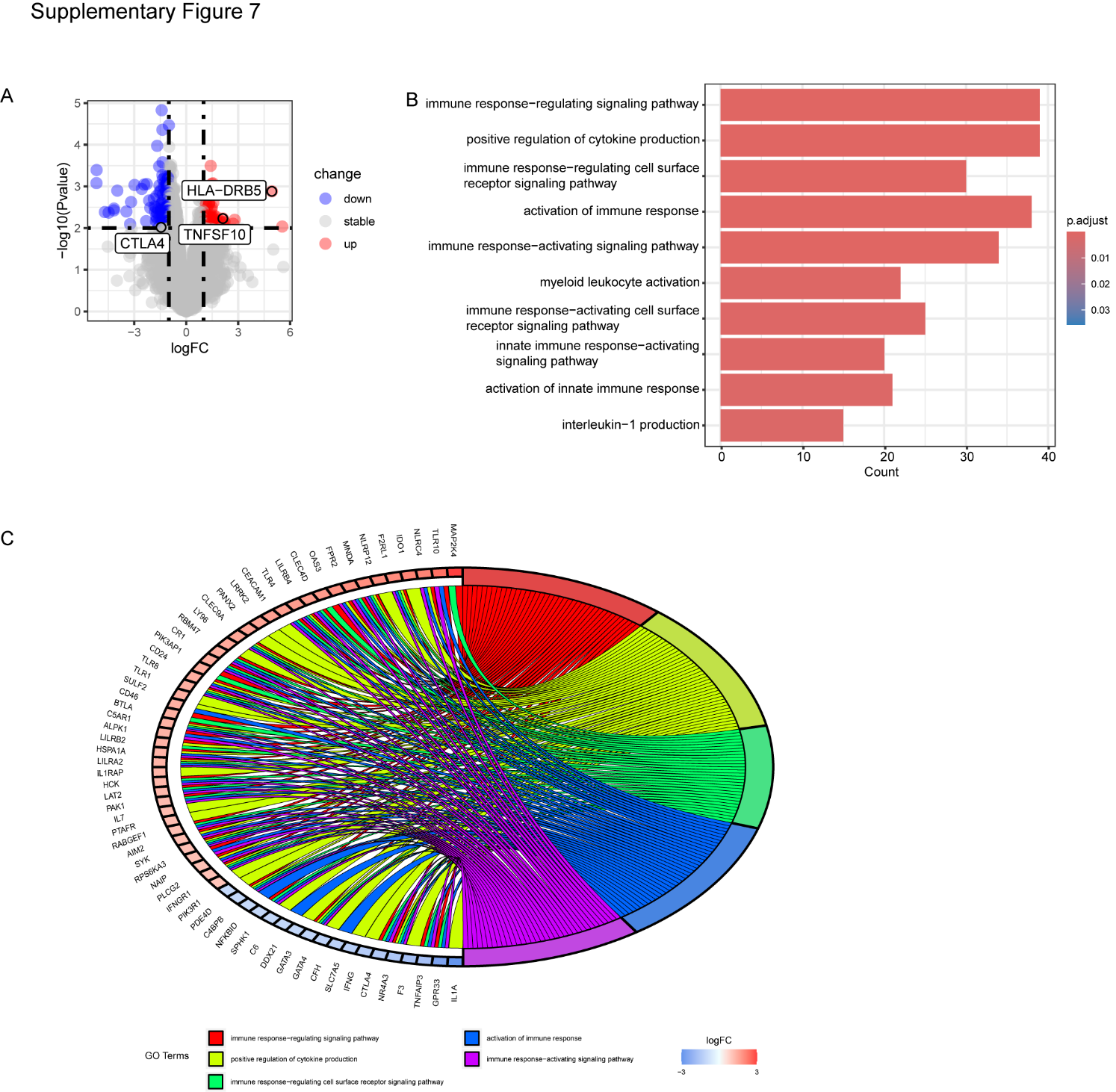


**Supplementary Figure 7.** Analyzing bulk transcriptomics data from the GEO database. (A) Volcano plots showing DEGs (Fold change>2, adjusted p-value<0.05) of PV vs. HC. (B) Barplot of GO terms enrichment analysis. (C) The circle graph of GO analysis.

**
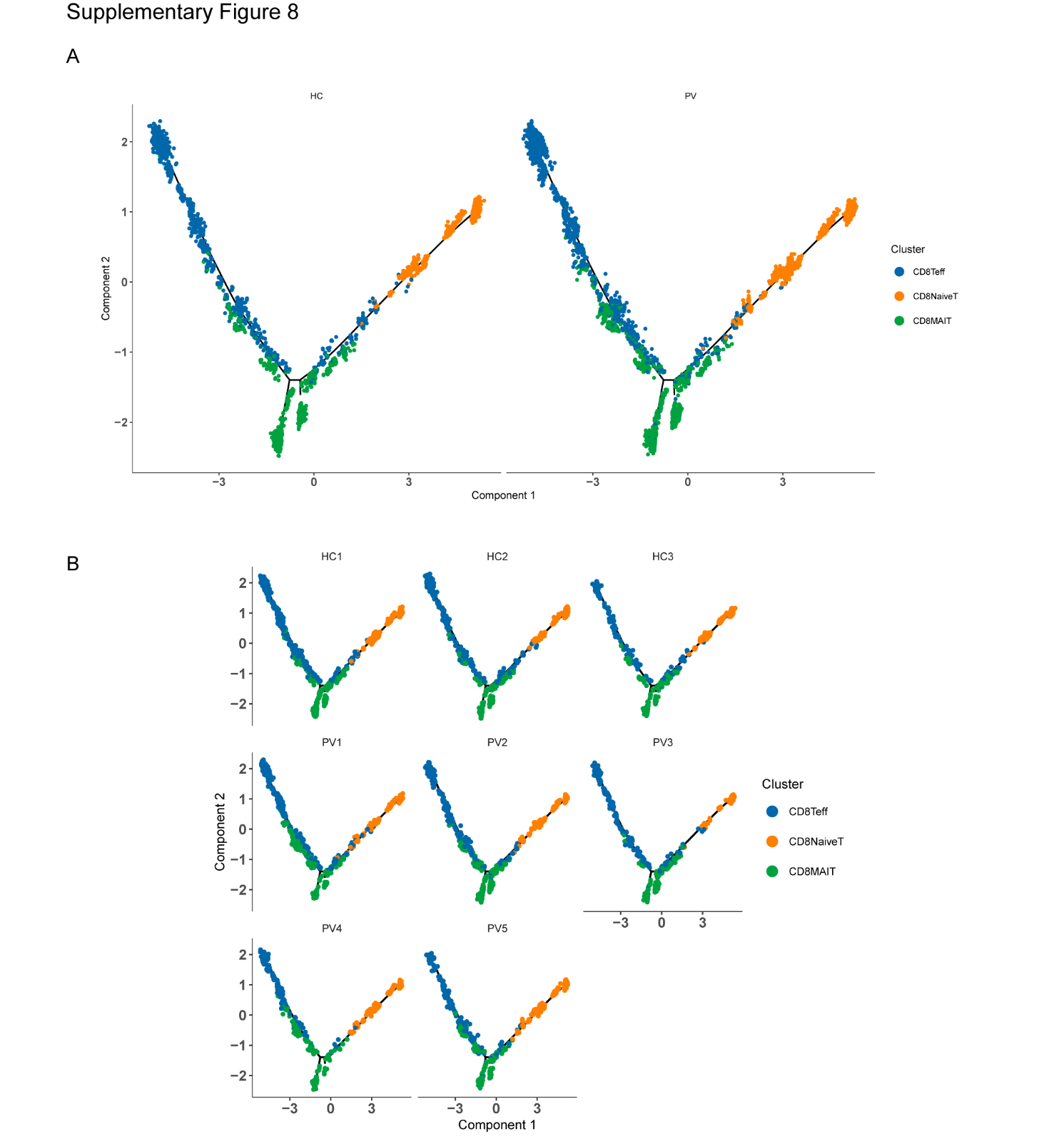
**

**Supplementary Figure 8.** Analysis of CD8+ T cells differentiation trajectories. Unsupervised transcriptional trajectory from Monocle, colored by groups (A) and samples (B).

# Supplementary Tables

**Supplementary Table 1.** Clinical information

**Supplementary Table 2.** Sample indicators; Number of cells in each cell subpopulation, proportion

**Supplementary Table 3.** Significant differentially expressed genes in T and NK cells

**Supplementary Table 4.** GO functions pathway analyses in T and NK cells

**Supplementary Table 5.** Significant differentially expressed genes in B cells

**Supplementary Table 6.** GO functions pathway analyses in B cells

**Supplementary Table 7.** Significant differentially expressed genes in monocytes

**Supplementary Table 8.** GO functions pathway analyses in monocytes

**Supplementary Table 9.** Significant differentially expressed genes in DCs

**Supplementary Table 10.** GO functions pathway analyses in DCs

**Supplementary Table 11.** Significant differentially expressed genes in neutrophils

**Supplementary Table 12.** GO functions pathway analyses in neutrophils
